# Design of MRI structured spiking neural networks and learning algorithms for personalized modelling, analysis, and prediction of EEG signals

**DOI:** 10.1038/s41598-021-90029-5

**Published:** 2021-06-08

**Authors:** Samaneh Alsadat Saeedinia, Mohammad Reza Jahed-Motlagh, Abbas Tafakhori, Nikola Kasabov

**Affiliations:** 1 Iran University of Science and Technology, Tehran, Iran; 2 Iranian Center of Neurological Research, Tehran University of Medical Sciences, Tehran, Iran; 3 School of Engineering, Computing and Mathematical Sciences, Auckland University of Technology, Auckland, New Zealand; 4 George Moore Chair of Data Analytics, University of Ulster, Londonderry, UK

**Keywords:** Computational models, Computational neuroscience, Data integration, Image processing

## Abstract

This paper proposes a novel method and algorithms for the design of MRI structured personalized 3D spiking neural network models (MRI-SNN) for a better analysis, modeling, and prediction of EEG signals. It proposes a novel gradient-descent learning algorithm integrated with a spike-time-dependent-plasticity algorithm. The models capture informative personal patterns of interaction between EEG channels, contrary to single EEG signal modeling methods or to spike-based approaches which do not use personal MRI data to pre-structure a model. The proposed models can not only learn and model accurately measured EEG data, but they can also predict signals at 3D model locations that correspond to non-monitored brain areas, e.g. other EEG channels, from where data has not been collected. This is the first study in this respect. As an illustration of the method, personalized MRI-SNN models are created and tested on EEG data from two subjects. The models result in better prediction accuracy and a better understanding of the personalized EEG signals than traditional methods due to the MRI and EEG information integration. The models are interpretable and facilitate a better understanding of related brain processes. This approach can be applied for personalized modeling, analysis, and prediction of EEG signals across brain studies such as the study and prediction of epilepsy, peri-perceptual brain activities, brain-computer interfaces, and others.

## Introduction

To measure and to analyze brain activities, MRI^1^, EEG^2–8^, and functional MRI (fMRI)^9^ are widely used for the diagnosis and treatment of diseases such as epilepsy^10^, for the prediction of brain surgery outcomes^12^, human muscle activity^13^, psychological analyses^6,8,14,17,34^, to mention only a few. The study of biological signals, which contain information about the activity and structure of the brain, has received much attention from researchers^3,10^ but still few studies combine data such as EEG and MRI to enhance reliability, accuracy and interpretability of the models^14^.

Analysis and prediction of bio-signals, due to their unique features and complexities, has always been a challenging task^15,16^. Diagnosis of any abnormal changes in the brain may indicate a disorder^14^, therefore awareness of the neuronal behavior along with the biomechanical structure can be remarkably effective^2^. Multimodal brain data, such as EEG and MRI have been collected in many studies^1,14^ and the challenge now is to develop computational methods and tools that integrate these data for a better understanding of brain processes and for a better prediction of personal events^1–3,20,21,23^.

Biologically inspired spiking neural networks (SNN) emerge as suitable techniques for modelling spatio-temporal brain data (STBD) such as EEG^3,17,21,22^, or fMRI^9,21^, or fMRI and DTI^18,21^. A brain-inspired SNN architecture, called NeuCube, to model STBD, has already been introduced^15^ and explored on single source brain data, such as EEG^3,17,21–23^, EMG^19^, fMRI^9,21^ or even on a combination of fMRI and DTI data^18,21^. The basic structure of this architecture consists of a spike encoding module, a 3D SNNr reservoir module structured according to a general brain template such as Talairach or Montreal Neurological institute (MNI), and an output regression or classification module. In the first step, the bio-signal is encoded as a spike train using spike encoding algorithms and then, the generated spike sequences are entered into the SNNr for unsupervised learning using the spike-time dependent plasticity rule (STDP)^15^. The outputs of the SNNr neurons are connected to a SNN-based classifier/regressor trained in a supervised mode^11,21^. The connection weights in the SNNr are updated according to an unsupervised STDP learning algorithm, which makes the network more biologically plausible, but the quality of its performance depends strongly on the encoding stage and the initial connection weights in the SNNr that are set following a small-world connectivity model^15,21,25^.

The paper addresses some new challenges in this field, namely:

- Instead of using general brain templates, we propose to use a personal MRI data for pre-structuring a personal SNNr model, MRI-SNNr, that could lead to a better analysis of personalized EEG data and better accuracy of EEG signal prediction.
- Developing new learning algorithms for EEG signals in a MRI-SNNr, based on real value EEG data, avoiding encoding the data into spike sequences.
- Using the models from above to predict brain signals in areas not measured and not used for training the models.
- Developing new methods for the analysis and interpretation of trained MRI-SNNr for a better understanding of the data and the personal brain processes.

The proposed here 3D MRI-SNNr architecture uses the Izhikevich neuronal model^16^ allowing for continuous EEG data to be processed in the SNNr and to avoid a preliminary spike encoding process. Using the Izhikevich neuron requires solving two differential equations governing the membrane processes and membrane potential of neurons in a model. These equations are:

1 $$\frac{dv}{dt}=0.04{v}^{2}+5v+140-u+I$$

$$\frac{du}{dt}=a \left\{bv-u\right\}$$

$${\mathrm{if}} \, v\ge 30 mV, {\mathrm{Then}}\; v\leftarrow c, u\leftarrow u+d$$

where: $v$ is the membrane potential; $I$ denotes the input current to the neuron as a continuous value; $u$ represents recovery current; a, b, c and d are constants, affecting the neuronal spike behavior. The voltage is in millivolts, and the time is in milliseconds.

The article is organized as follows: The second section introduces the proposed method and MRI-SNNr architecture for personalised modelling along with the learning algorithms. “Results and discussions” presents simulation results of the performance of two MRI-SNNr models when compared with the existing NeuCube model and with univariate EEG signal prediction method. “Discussions and Conclusion” suggests possible applications of the proposed method and directions for further research.

## The proposed method and MRI-SNNr architecture for personalised modelling and learning algorithms

### The method and the overall architecture

**Figure 1:**
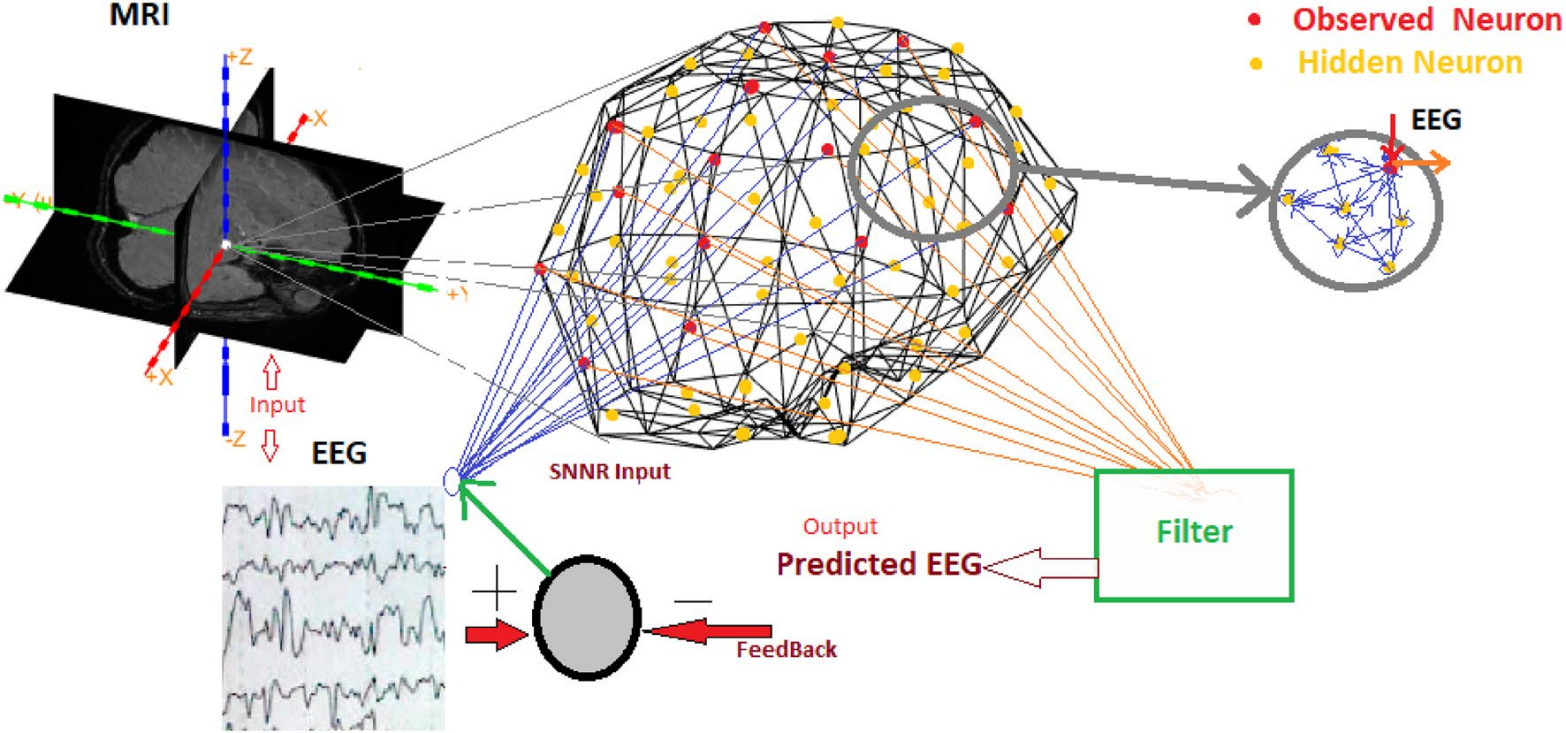
A schematic diagram of the proposed MRI-SNNr architecture for EEG signal modelling and prediction using personalized MRI data for structuring the SNNr (“hidden” neurons, in yellow,) and EEG input data for training the SNNr (input/output neurons, also called observed neurons, in red).

**Figure 2:**
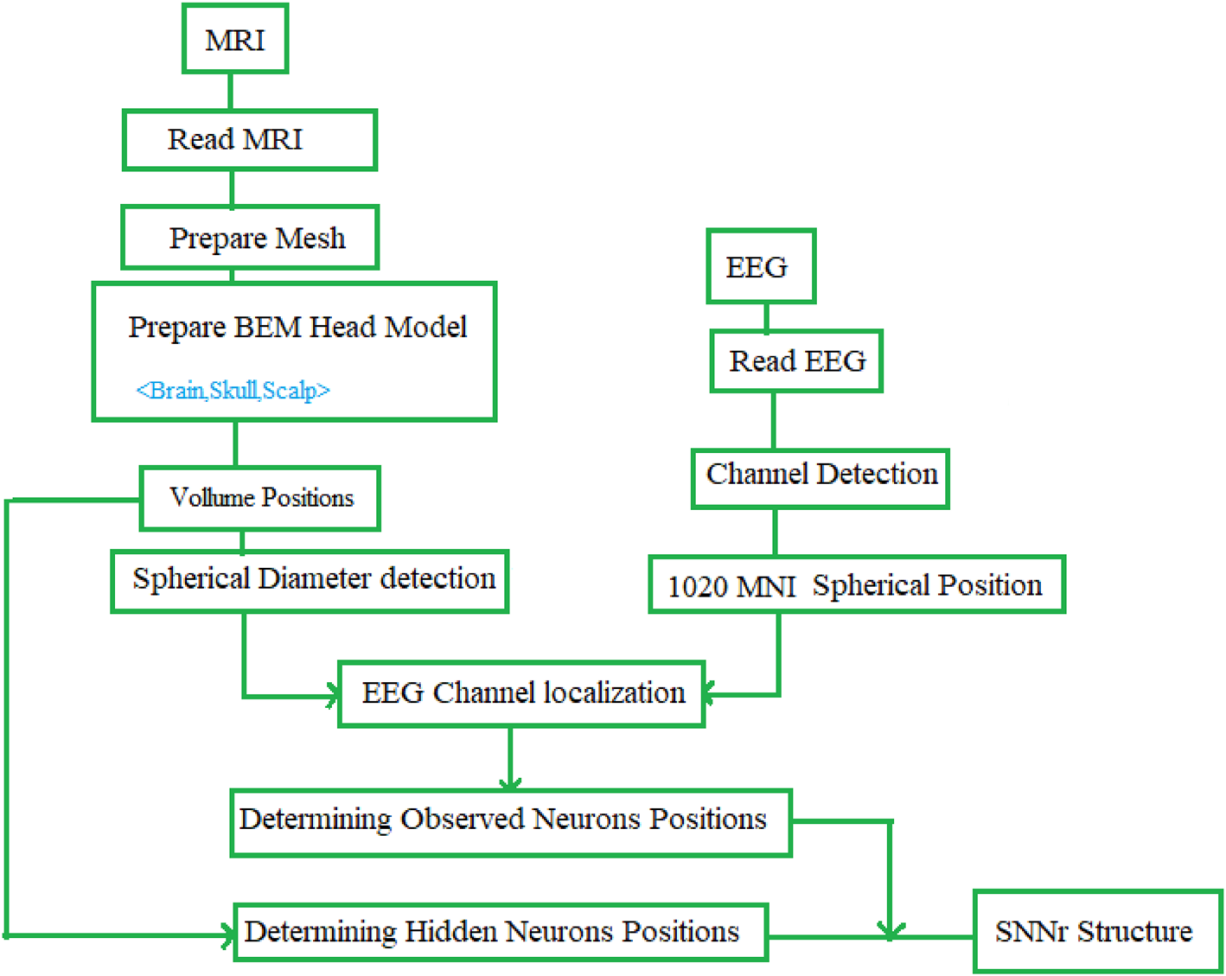
The Algorithm of Combining MRI and EEG data to calculate the positioning of the neurons in the 3D MRI-SNNr structure.

The proposed MRI-SNNr architecture consists of a SNNr and Output module (Fig. 1). The SNNr is structured according to MRI personal data, consisting of interconnected observed (input/output) and hidden neurons. The boundary element method (BEM)^26^ using FieldTrip tool^27^ and MATLAB software to localize brain activity are used to structure the 3D-SNNr using a personal MRI image data. The 3D locations of the extracted points from the Calculated-Volume-mesh grid are assigned to the spatial positions of spiking neurons in a 3D SNNr. The observed neurons are positioned proportional to the spatial locations of the EEG channels providing input information^15^. Figure 2 presents the algorithm for the spatial localization of the spiking neurons in the 3D SNNr following spatial information from both MRI and EEG data.

After structuring the MRI-SNNr, EEG data are used to train it. The connection weights between the spiking neurons are first initialized using small world connectivity rule^15,21,25^ as it is expected a higher impact of neighboring neurons so as to reduce the deviation of the normalized output EEG signal from the real EEG signal, described in the next section. In this paper, neural synaptic connection weights of the observed neurons are updated based on gradient descent learning rule.

The regulator/classifier module has the task of normalizing and filtering (0 ~ 60 or 70 Hz)^28,29^ the output data from the observed neurons to produce continuous value output signals (Fig. 1).

In this framework, it is presumed that there is a bi-directional link between neurons in a neighborhood, and each neuron output is connected to the same neuron as a feedback. ${N}_{i}$ denotes the neighborhood of i_th_ neuron as follows:

2 $${N}_{i}=\left\{j:{D}_{ij}\le T\right\}$$

where T is the maximum spatial distance of two neurons that are chosen depending on the problem. Here T = 70 mm, and ${D}_{ij}$ is the distance between neuron i and j. In the case of *i* = *j,* feedback from the output of the neuron to its input occurs**.**

Similar to^9^ initial bi-directional connection weights w_ij_ = w_ji_ = 0 between neighboring neurons i and j in the defined above SNNr are set before training of the SNNr with EEG data (see also Eq. 2):

3 $${w}_{ij}=\left\{\begin{array}{l}\begin{array}{ll}wji=0&\quad \quad  j\in {N}_{i}\end{array}\\ \begin{array}{ll}no \; connection &\quad j\notin {N}_{i}\end{array}\end{array}\right.$$

The connection weights of the observed (input/output) neurons are modified with the use of the presented below new GDR learning algorithm. The connection weights of the hidden neurons are modified during learning of EEG data using the STDP rule and after learning, only the strongest connection between every two neurons is kept as suggested in^9^.

The learning algorithms for both hidden and observed neurons in the MRI-SNNr are introduced in the next section.

### The proposed learning algorithms for the MRI-SNNr

We propose that the connection weights of the observed neurons are modified using the gradient descent rule (GDR) as described further below and the connection weights of the hidden neurons are modified with the use of the spike-time dependent plasticity (STDP) learning rule. The key motive of the GDR and STDP combination is to reduce the dependency of SNN on spike encoding algorithms, as GDR uses real value data, and to preserve biological plausibility still using STDP for spiking information processing in the hidden neurons. For completeness, both variants of continuous and spike inputs to the models are implemented and their performance is compared as in some studies only spike information is available.

The hidden neurons are updated according to the STDP learning rule^15,21^:

4 $$\Delta wij=\left\{\begin{array}{l} {A}_{+}\mathrm{exp}\left(\frac{\Delta t}{{\tau }_{+}}\right)  \quad \quad\,\,\, \; if\;\;  \Delta t\ge 0 \\ {-A}_{-}\mathrm{exp}\left(-\frac{\Delta t}{{\tau }_{-}}\right)  \quad if \;\; \Delta t < 0\end{array}\right.,$$

where $\Delta {w}_{ij}$ is the synaptic weight change in ${N}_{i}$ and Δt is the time difference between the spike time of the ith neuron and the postsynaptic neuron j. A_+_ and A_-_ are the maximum values of synaptic modification in the two directions and τ _+_ and τ_-_ represent the time constant of permissible changes in synaptic weights. The method for the modification of the observed-neuron-synaptic weights is presented below.

With regard to Eq. (1) and the relationship between membrane potential and excitation input current, this paper proposes a new formula for the calculation of the input current ${I}_{i}$ to the ith observed neuron of the Izhikevich type:

5 $${I}_{i}=\left(\sum_{{j\in N}_{i}}{w}_{ij}\frac{d{v}_{j}}{dt}+\frac{d{W}_{ij}}{dt}{v}_{j}\right)\left(1-{\mathit{tan}h\left(\sum_{{j\in N}_{i}}{w}_{ij}{v}_{j}\right)}^{2}\right)+\frac{d{\theta }_{i}}{dt},$$

where ${w}_{ij}$ is the synaptic weight between neurons i and j and $\frac{d{\theta }_{i}}{dt}$ is the *leakage current expression* of the ith neuron.

In fact, the suggested Eq. (5) can mitigate the calculation cost of the error backpropagation process ahead, while the effect of the neural activity of adjacent neurons is considered. In the other word, this paper has proposed Eq. (5) as the input current adaptation law with the aim of creating of linear algebraic summation relationship among membrane potentials of neurons in a neighborhood so as to reduce the cost of gradient descent calculation, while preserving the biological concept of the neural network by means of utilizing Izhikevich-type neurons.

The relationships for the observed neurons are rewritten in matrices form as follows:

6 $$I=\left(W\frac{\partial V}{\partial t}+\frac{\partial W}{\partial t}V\right)\left(\overrightarrow{1}-{\mathrm{tanh}({W}^{T}V)}^{2}\right)+\frac{\partial \theta }{\partial t},$$

where the parameters are described as below:

7 $$ \begin{gathered} V = \left[ {v_{i} } \right], \, \quad I = \left[ {I_{i} } \right],\quad \frac{\partial \theta }{{\partial t}} = \left[ {\frac{{d\theta_{i} }}{dt}} \right], \, \quad i = 1, \ldots, N \hfill \\ W = \left[ {W_{i} } \right]_{{N_{ob} \times N}}, \, \quad W_{i} = \left[ {w_{i1 } \cdots w_{ij} \cdots w_{iN} } \right], \quad i \in N_{ob} , j = 1, \ldots , N \hfill \\ \end{gathered} $$

and if $j\notin {N}_{i}$ then ${w}_{ij}=0$, where $N$ is the total number of reservoir neurons in the SNNr and ${N}_{ob}$ is the set of observed neurons.

A more general form of Eq. (1) is considered as the following:

8 $$\frac{\partial V}{\partial t}=g\left(V\right)+I$$

Then, considering $G\left(V\right)=\int g\left(V\right)dt$, $F\left(v\right)=\int Idt=\mathrm{tanh}(WV)+\theta$, on case of zero initial conditions, the neuronal membrane potential is calculated as:

9 $$V=G\left(V\right)+F\left(V\right)=G\left(V\right)+\mathrm{tanh}(WV)+\theta $$

The cost function J (t) at time t is defined as:

10 $$J(t)=\frac{1}{2}{E}^{T}E, \, \quad  E={\left[{e}_{i}(t)\right]}_{{N}_{ob}\times 1},\quad i\in {N}_{ob}$$

where ${e}_{i}\left(t\right)$ is the normalized ith neuron output error at time t, denoted by ${v}_{{n}_{i}}=\frac{{v}_{i}(t)}{{\Vert V(t)\Vert }_{2}}$, from the output of the EEG channel corresponding to the location of that neuron, represented by ${v}_{{d}_{i}}(t)$.

11 $${e}_{i}(t)={ v}_{{d}_{i}}(t)-{v}_{{n}_{i}}(t)$$

Substitute Eq. (11) into Eq. (10), the following equation is obtained:

12 $$J\left(t\right)=\frac{1}{2}{\left({V}_{d}-\left(\frac{1}{{\Vert V(t)\Vert }_{2}}\right){V}_{ob}\right)}^{T}\left({V}_{d}-\left(\frac{1}{{\Vert V(t)\Vert }_{2}}\right){V}_{ob}\right),$$

where $${V}_{d}={\left[{ v}_{{d}_{i}}(t)\right]}_{{N}_{ob}\times 1}$$ and $${V}_{ob}={\left[{v}_{i}(t)\right]}_{{N}_{ob}\times 1}$$.

Accordingly, weight modifications for the observed spiking neurons are determined based on the GDR:

13 $$ \frac{\partial W}{{\partial t}} = - \frac{\partial J}{{\partial W}} = - \left( {\frac{\partial J}{{\partial E}} \times \frac{\partial E}{{\partial V_{ob} }} \times \frac{{\partial V_{ob} }}{\partial F} \times \frac{\partial F}{{\partial W}}} \right) = - W^{T} \times \left( {\overset{\lower0.5em\hbox{$\smash{\scriptscriptstyle\rightharpoonup}$}} {1} - \tanh \left( {W^{T} V} \right)^{2} } \right)\left( {E \times \frac{ - 1}{{{\Vert V \left( t \right)\Vert}_{2} }} \times 1 \times V^{T} } \right) = \frac{1}{{{\Vert V\left( t \right) \Vert}_{2} }}W^{T} \times \left( {\overset{\lower0.5em\hbox{$\smash{\scriptscriptstyle\rightharpoonup}$}} {1} - \tanh \left( {W^{T} V} \right)^{2} } \right) \times E \times V^{T} $$

Thus, the rule of updating the observed neuronal synaptic weights concerning the effect of neighboring neurons is as follows:

14 $${\Delta {w}_{ij}}_{ob}=\left\{\begin{array}{l}\begin{array}{cc}{{{W}_{ij}}_{ob}}^{T}\times (1-{\mathit{tan}h\left({{W}_{ij}}_{ob}{v}_{j}\right)}^{2})\left(\frac{1}{{\Vert V\left(t\right)\Vert }_{2}}{e}_{i}\times {{v}_{j}}^{T}\right){T}_{s}& j\in {N}_{i}\end{array}\\ \begin{array}{ll}0&\quad\quad  j\notin {N}_{i}\end{array}\end{array}\right.$$

where ${T}_{s}$ is the simulation time step. The *leakage current expression* in the update law is calculated as:

15 $$\frac{\partial \theta }{\partial t}=-\frac{\partial J}{\partial \theta }=-\left(\frac{\partial J}{\partial E}\times \frac{\partial E}{\partial {V}_{ob}}\times \frac{\partial {V}_{ob}}{\partial \theta }\right)=-\left(E\times \frac{-1}{{\Vert V\left(t\right)\Vert }_{2}}\times 1\right)=\frac{1}{{\Vert V\left(t\right)\Vert }_{2}}E$$

Concerning Eqs. (4) and (14), combination of STDP and GDR, as a reinforcement learning strategy, is described as:

16 $$\Delta {w}_{ij}=\left\{\begin{array}{ll}\begin{array}{ll}{{w}_{ij}}^{T}\times (1-{\mathit{\text{tan}}h\left(\sum_{j}{w}_{ij}{v}_{j}\right)}^{2})\left(\frac{1}{{\Vert V\left(t\right)\Vert }_{2}}{e}_{i}\times {{v}_{j}}^{T}\right){T}_{s}&\quad  i\in {N}_{ob}\end{array},\quad j\in {N}_{i}\\ \begin{array}{ll}{A}_{+}{e}^{-\left(\frac{\Delta t}{{\tau }_{+}}\right)} \times {u}_{s}\left(-\Delta t\right) &\quad \quad i\notin {N}_{ob}, j\in {N}_{i} \end{array}\\ \begin{array}{ll}0 &\quad \quad \quad  j\notin {N}_{i}\end{array}\end{array}\right.$$

where ${u}_{s}\left(-\Delta t\right)$ is a step function, defined as:

17 $${u}_{s}\left(\zeta \right)=\left\{\begin{array}{l}\begin{array}{ll}1&\quad  \zeta \ge 0\end{array}\\ \begin{array}{ll}0&\quad \zeta \end{array}<0\end{array}\right.$$

### Two realisations of the proposed MRI-SNNr architecture

**Figure 3:**
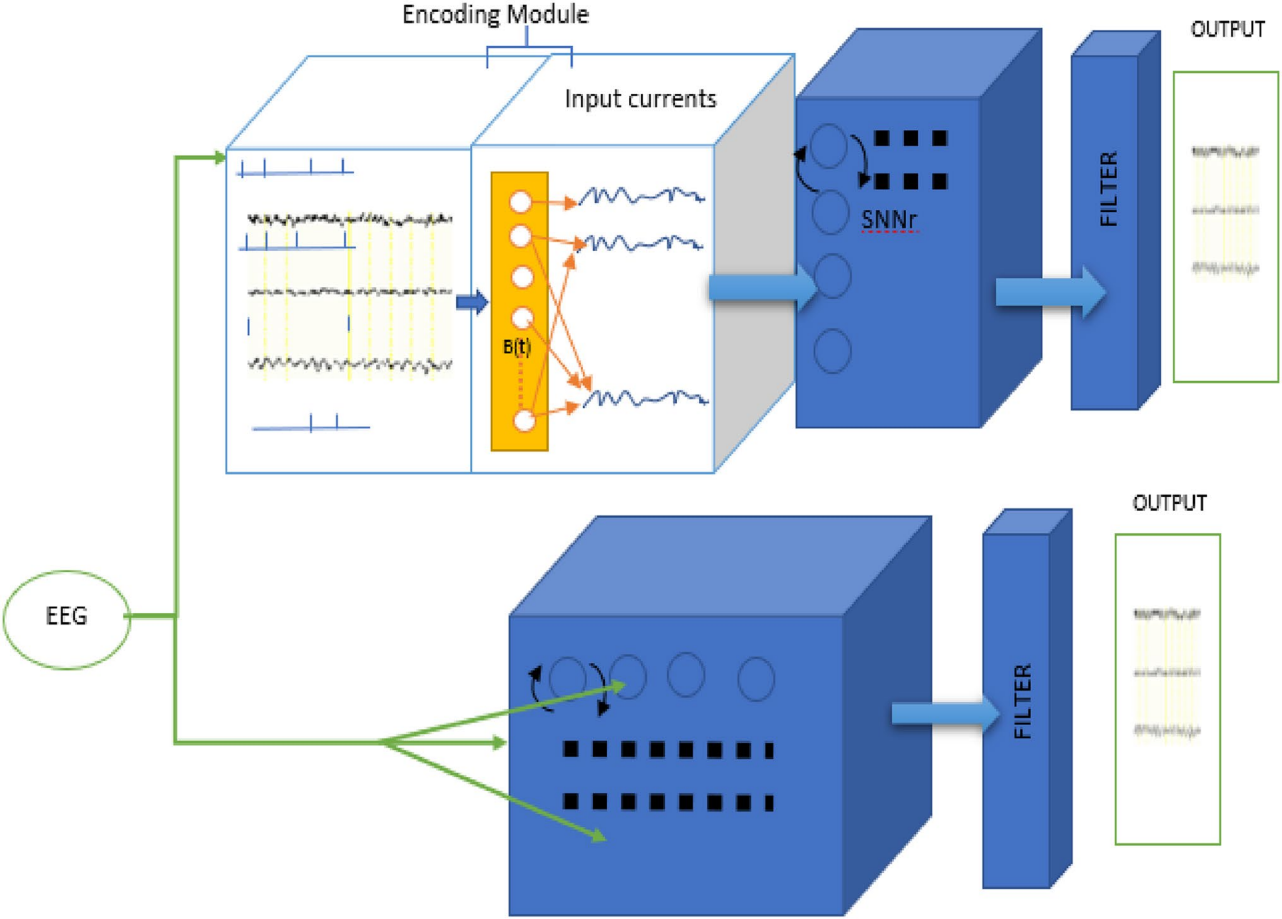
A schematic representation of the functionality of the created spike-based MRI-SNNr model (MRI-sSNNr) and continuous value MRI-SNNr (MRI-cSNNr) for modelling of EEG signals. In both realisations, same learning algorithms from “The proposed learning algorithms for the MRI-SNNr” are used.

The proposed MRI-SNNr architecture and learning algorithms are utilized here in two realizations: (a) continuous value EEG signals are used as inputs (MRI-cSNNr); (b) spike sequences are used as inputs as in many studies only spike sequence data is available (MRI-sSNNr) (Fig. 3).

In both realizations from Fig. 3, neurons in the MRI-SNNr are located using the same personalized MRI data. Parameters of the neurons and STDP learning are initialized according to Table 1. In the MRI-sSNNr, every spike sequence $X$ is converted into a continuous value signal using a baseline *B(t)* with regard to the method from^9^ and with regard to the raw input signal S(t), before the data is used for training the SNNr applying the same learning algorithm as in 2.2.

**Table 1:** Simulation Parameter Values for the Izhikevich type of neurons and STDP Parameters.

Excitatory neurons		Inhibitory neurons	
Parameter	Value	Parameter	Value
*A*	0.02	*A*	0.02 + 0.08*Random (0–0.08)
*B*	0.2	*B*	0.25–0.05*Random (0.2– 0.25)
*C*	− 5 + 25*Random (0–1)	*C*	Random (− 70 to − 60)
*D*	8–6*Random (0–1)	*D*	2
**STDP connection weights updating parameters**			
Parameter	Value	Parameter	Value
${\tau }_{+}$	5000	${A}_{+} \mathrm{and} A-$	$2\times {10}^{-3}$

18 $$\left\{\begin{array}{l}\begin{array}{ll}{t}_{m}={\mathrm{arg\;min}}_{t}S(t)& t\in \left\{{t}_{0},{t}_{1},{t}_{2},\dots ,{t}_{L}\right\}\end{array}\begin{array}{lll}& & \begin{array}{lll}& & \end{array}\end{array}\\ \begin{array}{ll}B\left({t}_{m}\right)=S({t}_{m})& \begin{array}{lll} & & \begin{array}{lll}& & \end{array}\end{array}\end{array}\\ \begin{array}{l}\begin{array}{ll}if \; S\left({t}_{i+1}\right)>B\left({t}_{i}\right)\to & B\left({t}_{i+1}\right)=\alpha S\left({t}_{i+1}\right)+\left(1-\alpha \right)B\left({t}_{i}\right) \begin{array}{ll}& \end{array} {t}_{i+1}\left(m\le i\le L\right),\alpha (\alpha \in \left[\mathrm{0,1}\right]) \end{array}\\ \begin{array}{ll} if\,\, S\left({t}_{i+1}\right)<B\left({t}_{i}\right)\to & B\left({t}_{i+1}\right)=S\left({t}_{i+1}\right)\end{array}\\ \begin{array}{ll}& \end{array}\end{array}\end{array}\right.$$

With regard to Eq. (1), and with the aim of real neural activity simulation corresponding to the input EEG signals, it is suggested that the general form of observed neuron’s potential be considered according to the desired potential and the actual EEG value of the corresponding channel (Eq. 19).

19 $${V}_{d}\left({t}_{i}\right)=\rho \left({t}_{i}\right){e}^{-\frac{{t}_{i}-{t}_{s}}{\sigma }} , \, \quad {t}_{s}\in X$$

where $X$ is obtained according to the following equations:

$$\begin{array}{ll}if\, S\left({t}_{i}\right)>B\left({t}_{i}\right)\to & X\cup \left\{{t}_{i}\right\}, i=\mathrm{0,1},2,\dots \end{array}$$

20 $$\begin{array}{ll}\left\{\begin{array}{l}\rho \begin{array}{ll}\left({t}_{i}\right)={V}_{EEG}\left({t}_{i}\right) &\quad { t}_{i}\in X\end{array} \\ \rho \begin{array}{ll}\begin{array}{ll}\left({t}_{i}\right)=\rho \left({t}_{i-1}\right) & \end{array}&\quad  if \, { t}_{i}\notin X\end{array}\end{array}\right.& \end{array}$$

where ${V}_{EEG}$ is the real input EEG signal. After calculating $E={V}_{d}-\left(\frac{1}{{\Vert V(t)\Vert }_{2}}\right){V}_{ob}$, the synaptic weights of the observed neurons are calculated based on the introduced algorithm in “The proposed learning algorithms for the MRI-SNNr”.

The next section compares the modelling performance of the proposed two MRI-SNNr models, the existing NeuCube model and a traditional univariate prediction method on two case studies of personal MRI and EEG data.

### Ethics declarations

The data, used to test the proposed methods in this paper, have been previously collected by other groups under ethical protocols and made available for research.

## Results and discussions

To compare the performance of the proposed in “The proposed method and MRI-SNNr architecture for personalised modelling and learning algorithms” two MRI-SNNr models with other methods for EEG signal modelling and prediction, two cases of study data have been used. In this regard, “Experimental results of the proposed personalized MRI-SNNr models compared with other EEG predictive modelling methods on the first case study EEG data”, “Would adding more neurons in the MRI-SNNr result in an increased accuracy of EEG signal prediction?”, “Can a MRI-SNNr model be used to predict signals at other locations of the SNNr corresponding to non-monitored brain areas?”, “Analysis of the experimental results” and “Neuronal activity analysis for a better understanding of brain activities” present analysis of experiments on the EEG data of the first case study, and “Experimental results on a second case study MRI and EEG data” presents experiments on the second case study data.

The first case study data were collected from the Epilepsy Long-term EEG Monitoring Center of the University Hospital (Imam Khomeini), approved by the Research Ethics Committee of the Neuroscience Research Institute of the Tehran University of Medical Sciences. It is carried out as part of a clinical study with the relevant guidelines and regulations, and the patient has given informed consent to this. The second dataset was collected from the freely accessible database http://eeg.pl/epi, and the experiment has been conducted during a routine procedure at the Warsaw Memorial Child Hospital. The patients with refractory epilepsy were selected for a pre-surgical study with the aim of removing the epileptogenic zone.

### Patients characteristics

Patient 1/Case study 1: Female, 28 years old, with epilepsy form of right mesial temporal lobe origin; recording more than 24 h.

Patient 2/Case study 2: Male, 9 years old, with severe epilepsy form of temporal lobe foci; recording 40 min.

Both case-study EEG data contain 15 channel-EEG signals with a 10–20 model of electrode location and pediatric montage A^29^. The first case-study data is raw EEG but the second one has been pre-processed by 0.5–70 Hz filter with additional 50 Hz notch filter. The under examination EEG channels are:

Patient 1: PO7, M2, P9, Oz, Pz, Fz, Cz, F8, F7, A2, A1, T4, T3, T5, and T6.

Patient 2: A1, A2, O1, O2, T3, T4, T5, T6, P3, Pz, P4, C3, Cz, C4, and F8.

Brain MRIs, containing T1 and T2 weighted brain scans with morphologic substrate of the epilepsy possess anatomical and dimensional sizes, respectively [260 320 80] and [512 512 176]. The grayscale—MRI—anatomy-measurement unit is *mm*.

### Experimental results of the proposed personalized MRI-SNNr models compared with other EEG predictive modelling methods on the first case study EEG data

Here, we compare the performance of the proposed two MRI-SNNr models from Fig. 3 with two other methods. The first one is a traditional univariate (a single EEG signal prediction) method, also called “random walk” that predicts the next time moment signal to be equal to the previous. The second model for comparison is NeuCube^15^, which uses spike encoded EEG signals for learning and the SNNr is structured using a general Talairach brain template rather than using a personalized MRI data. The NeuCube model has 1471 neurons in the SNNr, located at brain areas according to the used template^15^. The NeuCube software from (http://www.kedri.aut.ac.nz/neucube) is used.

The experimented MRI-SNNr has 300 Izhikevich neurons consisting of 15 observed (EEG channels) and 285 hidden neurons. Modeling properties of the network are set such that 20% of neurons are inhibitory and the rest are excitatory. In this paper, we considered that the time unit of Ts = 8 ms is appropriate to reconstruct the lower -than-70- Hz- frequency data, which includes all spike-based frequency bands. For a plausible comparison of the outputs of the models, both the input EEG signals and the network outputs are filtered simultaneously using a MATLAB–designed-Minimum-Ordered-Equripple-FIR filter (MOEF) with a 0–70 Hz pass frequency and 80 dB attenuation high frequency, containing the main frequency bands and eliminating drift (all filter parameters are listed in the Appendix) . The model parameter values used for the experiments are presented in Table 1.

To evaluate the performance of the proposed MRI-SNNr models and to compare it with other models, simulation is performed with the same EEG data. 70% of the data is used for training and 30% for testing.

**Figure 4:**
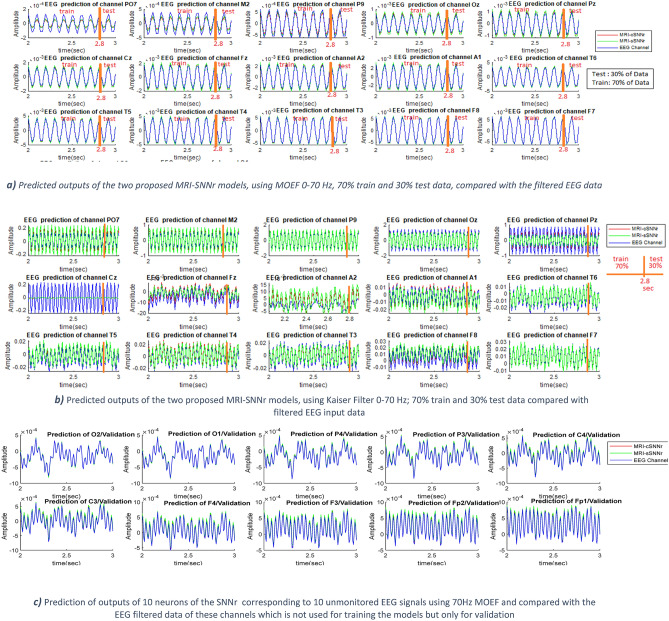
The blue line is the wave form of the real input signal after filtering for comparative analysis; the red line is the predicted output of the MRI-cSNNr and the green line—the predicted output of the MRI-sSNNr models: **(a) l**earning and testing of EEG data in the proposed MRI-SNNr models—MRI-cSNNr and MRI-sSNNr are compared with the 15 input EEG channel data after 0-70 Hz MOEF filtering ; **(b)** learning and testing of EEG signals in the two proposed MRI-SNNr models for 15 input channels using 0–70 Hz output Kaiser filter compared with the filtered input EEG signals. The learning process of the Cz channel in **(b)** failed; although Fp1 is not accurately learned in **(a)**, the experiment in **(a)** outperforms **(b)**. **(c)** Predicted signals at 10 neurons that correspond to unmonitored signals not used for training, while the models were trained on the measured EEG data from the 15 channels. It is seen that the prediction accuracy within the MRI-cSNNr structure is higher.

To evaluate the performance of the proposed MRI—SNNr models and to compare it with other models, simulation is performed with the same EEG data consisting of 500 samples measured every 8 ms. 70% of the data is used for training (350 samples, 2.8 s) and the future 30% for testing (150 samples, 1.2 s, but in Figs. 4 and 6 only the first 0.2 s are visualized). During training, input data are entered one by one and the observed neurons are trained to predict their next time output (in 8 ms) by using the output error and the GDR algorithm. During training, hidden neurons update their synaptic weights by using STDP law. During testing, there is no update of the connection weights of observed neurons. During the test procedure, no data samples are entered and the model predicts 1.2 s of the signal ahead. In this stage, only STDP is utilized in order to update the weights in the hidden neurons, unsupervised. In this regard, the observed neuron's output is measured and compared with the actual values to calculate the test error and all the indicated parameters in Table 2 has been calculated based on all 500 samples error.

**Table 2:** MSE,SD and P-Value Test Comparison of Several Methods.

Model/method Filter features	MSE (V^2^ × 10^–6^)						Max (SD (mV))	P-value
	15/15 channels (I/O) 0–60 Hz MOEF/100 neurons	15/15 channels (I/O) 0–70 MOEF /100 neurons	15/25 channels (I/O) prediction/0–70 Hz Moef /300 Neurons		15/25 channels (I/O) prediction/0–70 Hz Kaiser filtering/300 neurons	15/25 channels (I/O) prediction/0–70 Hz MOEF/400 neurons	15/25 channels(I/O) source prediction /0–70 Hz MOEF/300 neurons	15/25 channels(I/O) source prediction /0–70 Hz MOEF/300 neurons-significant level = 0.05
The proposed MRI-cSNNr	0.356	0.150	0.197		5.7	0.00015	0.0427	0.0359
The proposed MRI-sSNNr	1.23	0.52	0.72		12.4	0.00051	0.0831	0.0436
NeuCube^8^	0.87 (15 inputs,15 outputs)			0.88 (15 inputs, 16 outputs)			0.0517	0.0349
Univariate EEG predictive modelling	3.59						0.3811	0.0127

Prediction results for the two MRI-SNNr models are presented in Fig. 4a for each of the 15 EEG input channels, with 0-70 Hz output MOEF filter applied on both the outputs and on the input data for comparison of results. In order to work out the effect of the filter characteristics, Kaiser filter with 0–70 Hz frequency band and 80 dB attenuation at higher frequencies, has been imposed in Fig. 4b as the post-processor of raw EEG signals and also post-processor of outputs of the suggested network. A glance at Fig. 4 reveals that the shape of filtered outputs and inputs is vastly relevant to the sort of utilized filter and the other characteristics of the output filter such as frequency passband. The proposed continuous value-based MRI-cSNNr outperforms the spike-based MRI-sSNNr in terms of convergence, speed and prediction error in both a and b. With regard to the lower prediction error while utilizing the MOEF 0-70 Hz, subsequent simulations are performed with this filter. In a following section we will explain how such models can predict outputs at other neurons which correspond to unmonitored/ not measured area of the brain (Fig. 4c).

### Would adding more neurons in the MRI-SNNr result in an increased accuracy of EEG signal prediction?

Addressing this question, experiments are performed by examining the mean square error (MSE) of EEG prediction in both MRI-SNNr models with 100, 300 and 400 neurons. All parameters and initialization within the 400-neuron SNNr are similar to the 300-neuron SNNr in both structures. The MSE error of predicting EEG signals across all channels are shown in Table 2 for both structures. The estimated MSE for the 300-neuron SNNr models is not substantially different from the 100-neuron network MSE, while the estimated MSE for the 400-neuron SNNr models has dropped significantly. While increasing the number of neurons is not expected to have a linear effect, more neurons allow for more areas of the brain to be investigated. The determination of the appropriate values for the neuron parameters can influence the prediction accuracy, although the broad analysis of this issue is not the aim of this paper. The next simulations therefore proceed with 300 neurons and the same initialization.

### Can a MRI-SNNr model be used to predict signals at other locations of the SNNr corresponding to non-monitored brain areas?

As it is well accepted in the literature for focal epilepsy localization for example, the more EEG channel information is used, the higher the accuracy of focal localization is achieved^30,31^. On the other hand, there can be limitations in recording channel information and some hospitals can monitor only a limited number of channels, say around 32, simultaneously. Would a 3D MRI structured SNN model provide a possibility of predicting signals at other locations of the model in addition to the EEG channel locations (observed neurons)? If this is achieved, it may become possible to detect changes in other parts of the brain where no EEG channels are located. And this will be due to the use of MRI personal information to structure the SNNr.

In this section the performance of the two MRI-SNNr models from Fig. 3 to predict signals at SNNr locations that correspond to non- recoded EEG channel is evaluated. For this purpose, 15 channels of EEG signals are considered as inputs and 25 EEG channel locations in the 3D SNNr model are examined as outputs, including the additional 10 locations of hidden neurons that correspond to presumed non-observed 10 EEG channels. Figure 4c illustrates 10 EEG channels prediction performance to validate the estimation of non-recorded EEGs. The 10 continuous value signals, not used for training but evaluated for prediction, correspond to EEG channel positions O2, O1, P4, P3, C4, C3, F3, F4, Fp1, and Fp2 not used in the training data. MSE of the EEG signal prediction across all 10 new sites as well as 15 inputs’ prediction is shown in Table 2.

Proper convergence in the prediction of un-observed sources indicates the potential of this method in predicting unmonitored brain areas which constitutes the first study in this respect.

### Analysis of the experimental results

Table 2 presents some statistical analysis, entailing maximum Standard Deviation (SD) of prediction error of all predicted channels and the total mean square error (MSE)^31^ of EEG prediction of several methods when compared with the proposed MRI-SNNr methods for 0–60 Hz and 0–70 Hz output module filtering. The table shows that the proposed method for EEG signal modelling and prediction based on the proposed MRI-SNNr approach has resulted in several orders of magnitude better prediction accuracy. The following equation describes the MSE calculation:

21 $$MSE=Expectaion\left\{{\left(E\right)}^{T}\left(E\right)\right\}$$

where E is the normalized-one-step-ahead-prediction error vector, outlined within Eq. (10).

In order to create a suitable comparison between NeuCube and the novel proposed methods, the F3 channel is also considered as the unmonitored signal output of NeuCube. The experiment indicated that NeuCube has the capacity of predicting unmonitored signals as much as the suggested structure, but its' prediction error is higher.

Another set of experiments was conducted with 100 and 400 spiking neurons instead of 300 as reported in Fig. 4. Results of all experiments above are presented and validated in Table 2.

It can be seen from Table 2 that selection of the output filter parameters, such as type of the filter, stop band, pass band and attenuation, influence the accuracy of the models. Lowest error was registered for 400 spiking neurons in the SNNr when compared with 100 and 300. P-Value analysis, as another validation criterion, also indicates that both real data and predicted ones, are statistically analogous.

### Neuronal activity analysis for a better understanding of brain activities

**Figure 5:**
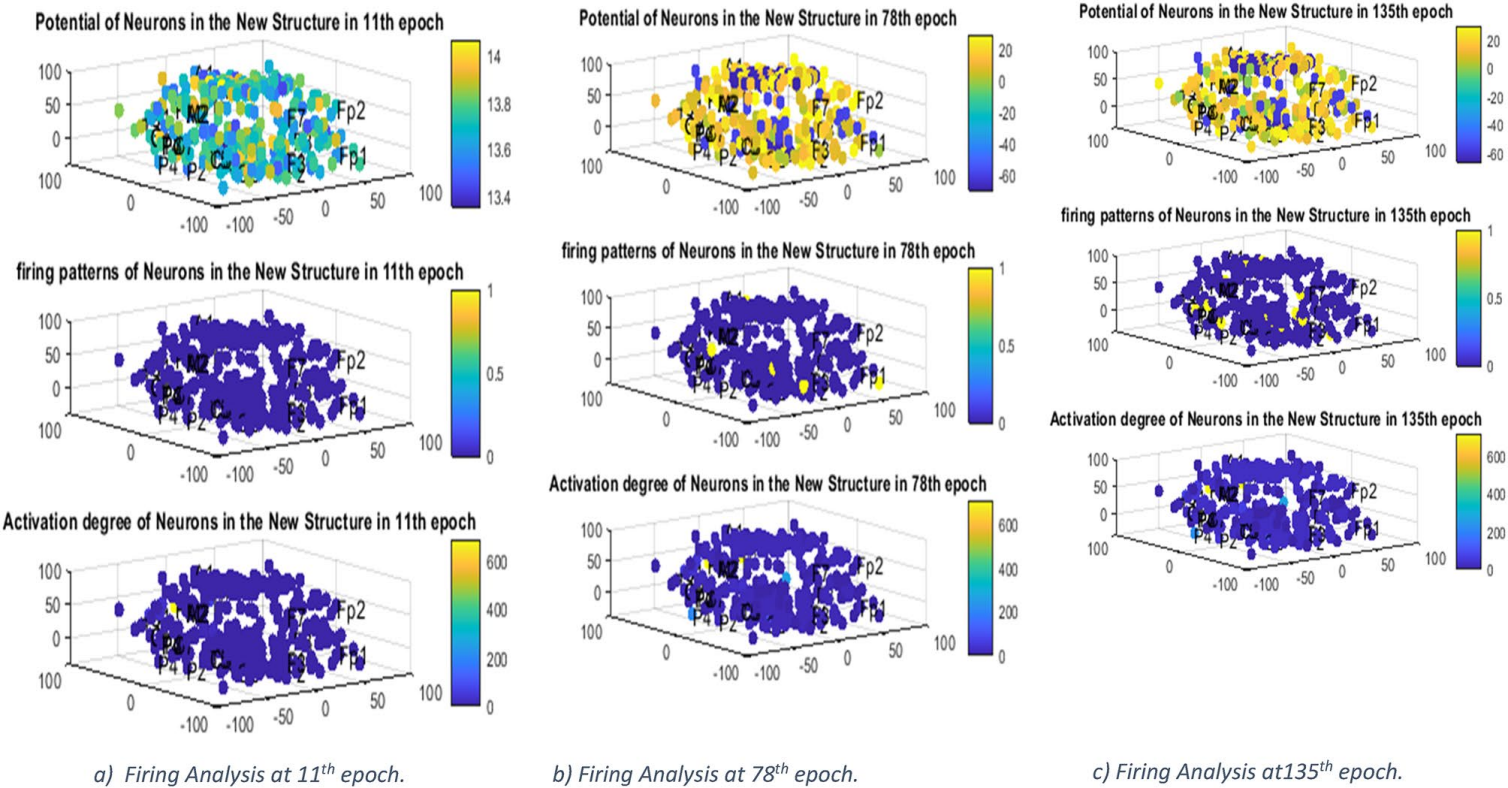
Distribution of membrane potentials of the neurons in the MRI-cSNNr (the first figure); firing patterns of the neurons (second figure) and activation degree of the spiking neurons (third figure) at: (**a) **11th epoch, **(b) **78th epoch, **(c)** 135th epoch of incremental training. The input neurons allocated for the corresponding EEC channels are labelled by the names of the channels.

Connection weights analysis of the MRI-cSNNr model could be used to better understand functions of the brain. In our model, the activation degree of each neuron is defined as suggest in^9^ by:

22 $${D}_{j}=\frac{{\sum }_{j}\left({w}_{ij}+{w}_{ji}\right)}{Number \, of \, neurons\, in\, {N}_{i}} j\in {N}_{i}$$

where ${w}_{ij}$ and ${w}_{ji}$ are the weights of bidirectional connections between neurons i and j which included negative and positive values. Figure 5 shows the degree of activation of each of the 3D neurons in the SNNr alongside the neuronal potential distribution and its firing pattern in 11, 78 and 135 epochs of incremental training of the MRI-SNNr models. epochs.

Here the EEG signals were reported from a patient undergoing hyperventilation (HV). The pattern of degree of activation converges after 100 epochs to a specific pattern, which can be further explored in future research when compared with a pattern produced by standard stimuli. Having studied some researches in the field of HV effects on epileptic EEG neural activity rhythms^4,5^ and owing to what can be inferred here, the degree of activation of the brain's central and parietal lobes under hyperventilation rises compared to other regions and the prediction of the signals in all measured regions have been accurately achieved as presented in the previous sub-sections.

### Experimental results on a second case study MRI and EEG data

**Figure 6:**
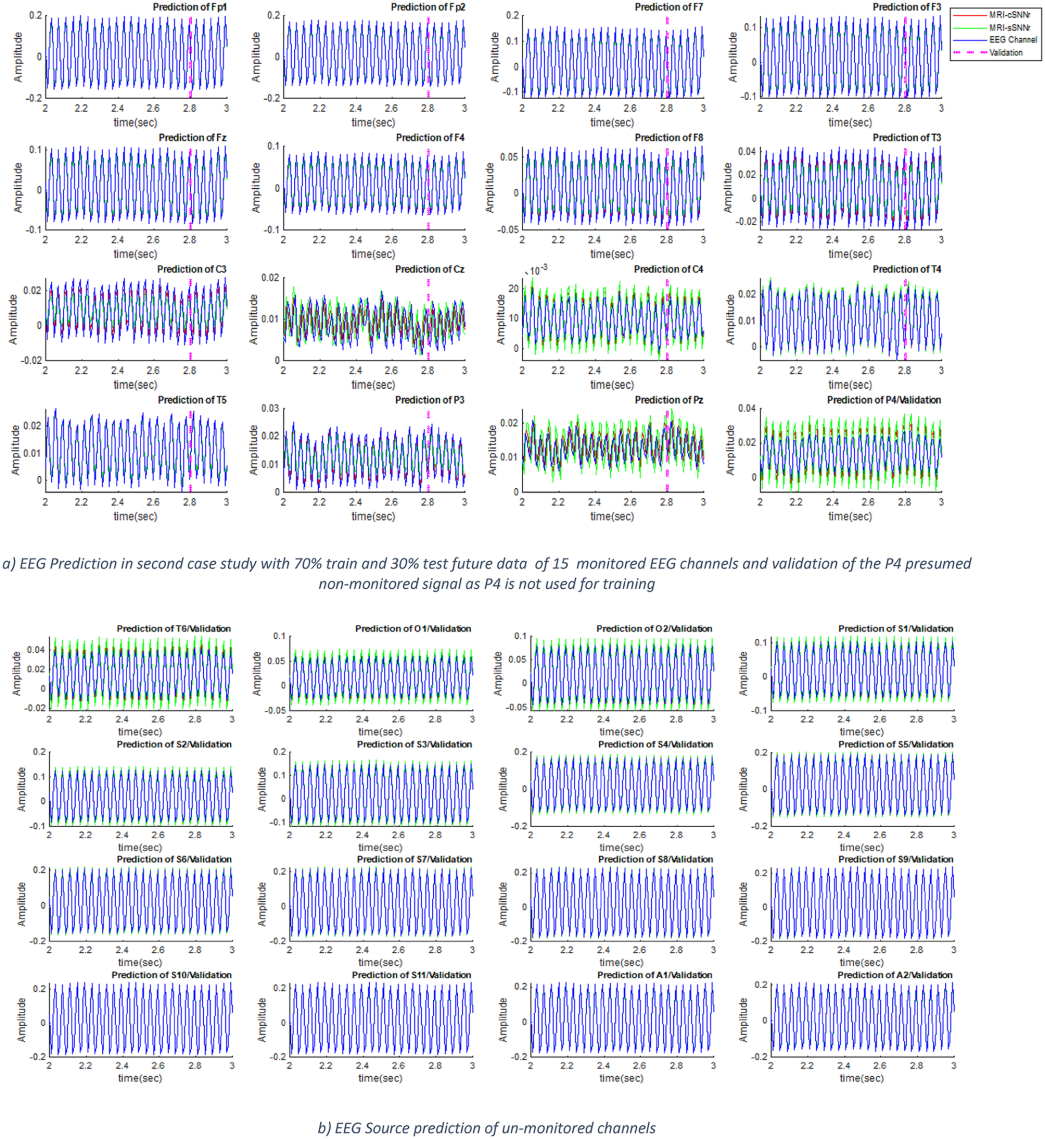
Predicted signals of second case study experiment at 32 locations (15 EEG channels and 17 other locations) **(a)** EEG Prediction of monitored 15 channels + the non- monitored P4 channel signal prediction along with the filtered input EEG data; **(b)** EEG source prediction of un-monitored 16 channels by MRI-cSNNr model (the red line) versus the real EEG signal after it is filtered (the blue line) and the spike-based MRI-sSNNr model (the green line). MRI-cSNNr indicates higher precision prediction in comparison to MRI-sSNNr.

In order to further validate the proposed method, EEG signal prediction test is developed for the second case study data in a similar way as for the first case study, with 15 monitored inputs, and 32 outputs, including 17 non-monitored channels (Fig. 6). The non-monitored channels are P4, T6, O1, O2, S1 ~ S11, A1, A2.

Figure 6 reveals that the suggested strategy is successfully able to predict EEG signals in both status, namely, 70% training and 30% test of monitored data or 100% testing of non-monitored EEG channels as a part of total data.

**Figure 7:**
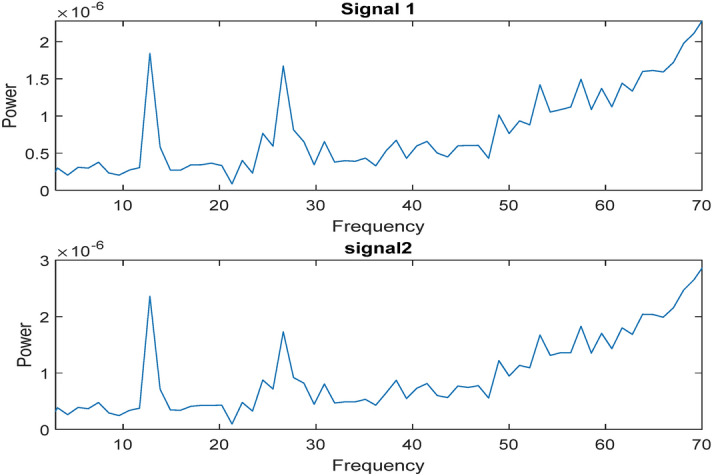
Power spectra of the input EEG Fp1 signal (signal 1) and the output signal from the corresponding neuron in the SNNr model, both filtered in the same way for a proper comparison of the output.

Figure 7 illustrates the similarity between the power spectrum of the input EEG FP1 signal (signal 1), after being filtered for comparison with the signal from the output module, also filtered. We can see that a similar power spectrum of the signal is produced by the model to the one of the input EEG signal which is also an indication for an accurate modelling result.

As it is shown in the above figures, although the signals in Fig. 4 and Fig. 6 may visually look similar to a sine wave, the frequency analysis shows that the recorded signals contain more widespread frequency bands rather than a mere sinusoidal wave. The wave signals for all channels in all experiments in this paper are visualized after applying the same filtering. Some of the wave forms may look similar to a sine form when perceived visually, but the spectrum analysis shows a complex frequency spectrums rather than a single frequency.

## Discussions and conclusion

In this paper, a new method is introduced for the creation of personalized SNN models, MRI-SNNr, for the analysis, prediction and understanding of EEG signals, where MRI data of a person is used to structure spatially a 3D SNN model. The 3D SNNr model consists of observed (input/output) neurons and hidden neurons. A new machine-learning algorithm is proposed for the MRI-SNNr to learn the EEG data using gradient descent learning rule for the observed Izhikevich neurons and STDP learning rule for the hidden neurons. Both continuous value inputs and spike inputs are considered in two separate versions of the proposed MRI-SNN architecture.

The presented preliminary experimental results on case study personal EEG data manifest a better performance of the proposed method applied on continuous value EEG signals rather than on spike encoded signals, both resulting in several orders of magnitude better modelling accuracy than a traditional statistical method and the NeuCube model^15^, which is structured according to a general brain template rather than a personal MRI information.

The learned neural connections in the MRI-SNNr, when using input data to the observed neurons to train a model, makes it possible to predict signals in other neurons of the model that correspond to non-measured (not observed) brain areas. This method can be interpreted for a better understanding of larger scale brain activity across applications. The strong performance of the MRI-SNNr in the prediction and reconstruction of signals of the SNNr corresponding to unmonitored EEG sources (data not used for training the models) suggests that this approach can be potentially used also for localizing critical brain regions. This is the world first research that demonstrates that a MRI pre-structured personalised SNN model trained on data from a number of EEG channels, can be used to predict the activity of other areas of the brain corresponding to EEG channels that are not used for training the model. The proposed method was illustrated on predictive learning and analysis of EEG data of the epileptic patients. Dynamic changes in EEG spatial–temporal patterns in different brain areas were discovered, which could be explained by transitions between epileptiform, preictal, and ictal events^28^. For the used case study of personal MRI and EEG data, it was found through analysis of the MRI-SNNr model that the right temporal region has the highest potential, which is consistent with the reported location of focal epilepsy in that area for this subject. The method was also illustrated on a second case study MRI and EEG data, and the high potential of hidden states of the brain functions would stem from the ability of 17 other non-monitored source prediction. The proposed method has the potential to be used for further study and prediction of epilepsy events^32,33^.This requires further investigation with the use of data from other individuals.

The proposed MRI-SNNr architecture and learning algorithms are of a generic type and could be potentially used across wide range of brain studies, such as peri-perceptual studies^34^, brain-computer interfaces^22^ and other^21^.

The accuracy of modeling depends on many parameters, related but not limited to, how MRI-SNNr is structured to map a personal MRI; what are the optimal parameters of learning in the SNNr; what are the optimal output functions and filter parameters. More research is planned to examine and optimize these parameters for a better personalized predictive modelling of EEG data. Representing spatio-temporal patterns of activities in the MRI-SNNr during learning in the form of spatio-temporal symbolic rules is also a challenging task that is also considered for future research by the team^13,21^.

## Supplementary Information

Supplementary Information.
