# Supplementary material for: Design of MRI structured spiking neural networks and learning algorithms for personalized modelling, analysis, and prediction of EEG signals
Source: Sci Rep. 2021 Jun 8;11:12064. doi: 10.1038/s41598-021-90029-5 (PMC8187669; doi:10.1038/s41598-021-90029-5)
Supplement: Supplementary file 1 — Supplementary Information. [file 41598_2021_90029_MOESM1_ESM.docx]

**Design of MRI Structured Spiking Neural Networks and Learning Algorithms for Personalized Modelling, Analysis, and Prediction of EEG Signals**

Samaneh Alsadat Saeedinia^1^, Mohammad Reza Jahed-Motlagh^1*^, Abbas Tafakhori^2^, Nikola Kasabov*^3,4^

* Contact persons

1: Iran University of Science and Technology, Tehran, Iran

2: Iranian Center of Neurological Research, [Tehran University of Medical Sciences](https://scholar.google.com/citations?view_op=view_org&hl=en&org=16806557757143553874), Iran

3: Auckland University of Technology, Auckland, New Zealand

4: University of Ulster, Londonderry, UK

______________________________________________________________________

**Corresponding Authors:**

^*1^Mohammad Reza Jahed -Motlagh

Professor of Computer Science

Phone: +98 21 73225306

 Email: [jahedmr@iust.ac.ir](mailto:jahedmr@iust.ac.ir)

and

^*3,4^ Nikola Kasabov (contact person for the publication)

^*3^ Professor, School of Engineering, Computing and Mathematical Sciences,

Auckland University of Technology; Phone: +64 9 921 9506; Email: [nkasabov@aut.ac.nz](mailto:nkasabov@aut.ac.nz)

^4^ George Moore Chair of Data Analytics, University of Ulster, UK; [nk.kasabov@ulster.ac.uk](mailto:nk.kasabov@ulster.ac.uk)

**Appendix.** Parameters of the used filter for filtering the model output signals and input signals for comparison as per Fig.1.

Hd = dsp.FIRFilter( ...

'Numerator', [-0.000139541582927379 -0.000207365984598939 ...

-0.000339007322766016 -0.000501185633741699 -0.00068420446474159 ...

-0.000871028896451993 -0.00103758153904352 -0.00115357708453446 ...

-0.00118480500235383 -0.0010962194509447 -0.000856372951093377 ...

-0.000442099383449745 0.000156616806859531 0.000932392631603575 ...

0.00185726166028893 0.0028815257949492 0.00393493705147475 ...

0.00493056352804387 0.00577122711680528 0.00635840242845638 ...

0.00660279055573616 0.00643558360252949 0.00581909344304093 ...

0.00475554981097269 0.00329270830271118 0.00152514742612678 ...

-0.000409600155770853 -0.00234024473470246 -0.00407593045169871 ...

-0.00542443018477447 -0.006212859974075 -0.00630882488917628 ...

-0.00563966344528029 -0.00420736338776815 -0.00209701371071784 ...

0.000522924939847415 0.00340947063618721 0.0062631542057572 ...

0.00875424650052982 0.0105552410694363 0.011376725895088 ...

0.0110031524848167 0.00932467529617034 0.00636137523147331 ...

0.0022766350501674 -0.00262270114841583 -0.00790059970866219 ...

-0.0130203002925458 -0.0173852555643109 -0.0203894338913031 ...

-0.0214727515123204 -0.020176588241793 -0.0161940305864881 ...

-0.00940969238308206 7.52622299691405e-05 0.0119360527605659 ...

0.0256367216681186 0.0404618120834439 0.055565148516195 ...

0.0700318961306696 0.0829486913261332 0.0934757322355358 ...

0.100914391079878 0.10476423297545 0.10476423297545 0.100914391079878 ...

0.0934757322355358 0.0829486913261332 0.0700318961306696 ...

0.055565148516195 0.0404618120834439 0.0256367216681186 ...

0.0119360527605659 7.52622299691405e-05 -0.00940969238308206 ...

-0.0161940305864881 -0.020176588241793 -0.0214727515123204 ...

-0.0203894338913031 -0.0173852555643109 -0.0130203002925458 ...

-0.00790059970866219 -0.00262270114841583 0.0022766350501674 ...

0.00636137523147331 0.00932467529617034 0.0110031524848167 ...

0.011376725895088 0.0105552410694363 0.00875424650052982 ...

0.0062631542057572 0.00340947063618721 0.000522924939847415 ...

-0.00209701371071784 -0.00420736338776815 -0.00563966344528029 ...

-0.00630882488917628 -0.006212859974075 -0.00542443018477447 ...

-0.00407593045169871 -0.00234024473470246 -0.000409600155770853 ...

0.00152514742612678 0.00329270830271118 0.00475554981097269 ...

0.00581909344304093 0.00643558360252949 0.00660279055573616 ...

0.00635840242845638 0.00577122711680528 0.00493056352804387 ...

0.00393493705147475 0.0028815257949492 0.00185726166028893 ...

0.000932392631603575 0.000156616806859531 -0.000442099383449745 ...

-0.000856372951093377 -0.0010962194509447 -0.00118480500235383 ...

-0.00115357708453446 -0.00103758153904352 -0.000871028896451993 ...

-0.00068420446474159 -0.000501185633741699 -0.000339007322766016 ...

-0.000207365984598939 -0.000139541582927379]);
